# Supplementary material for: Diverse motif ensembles specify non-redundant DNA binding activities of AP-1 family members in macrophages
Source: Nat Commun. 2019 Jan 24;10:414. doi: 10.1038/s41467-018-08236-0 (PMC6345992; doi:10.1038/s41467-018-08236-0)
Supplement: Supplementary file 1 — Supplementary Information [file 41467_2018_8236_MOESM1_ESM.pdf]

1 Supplementary Figures

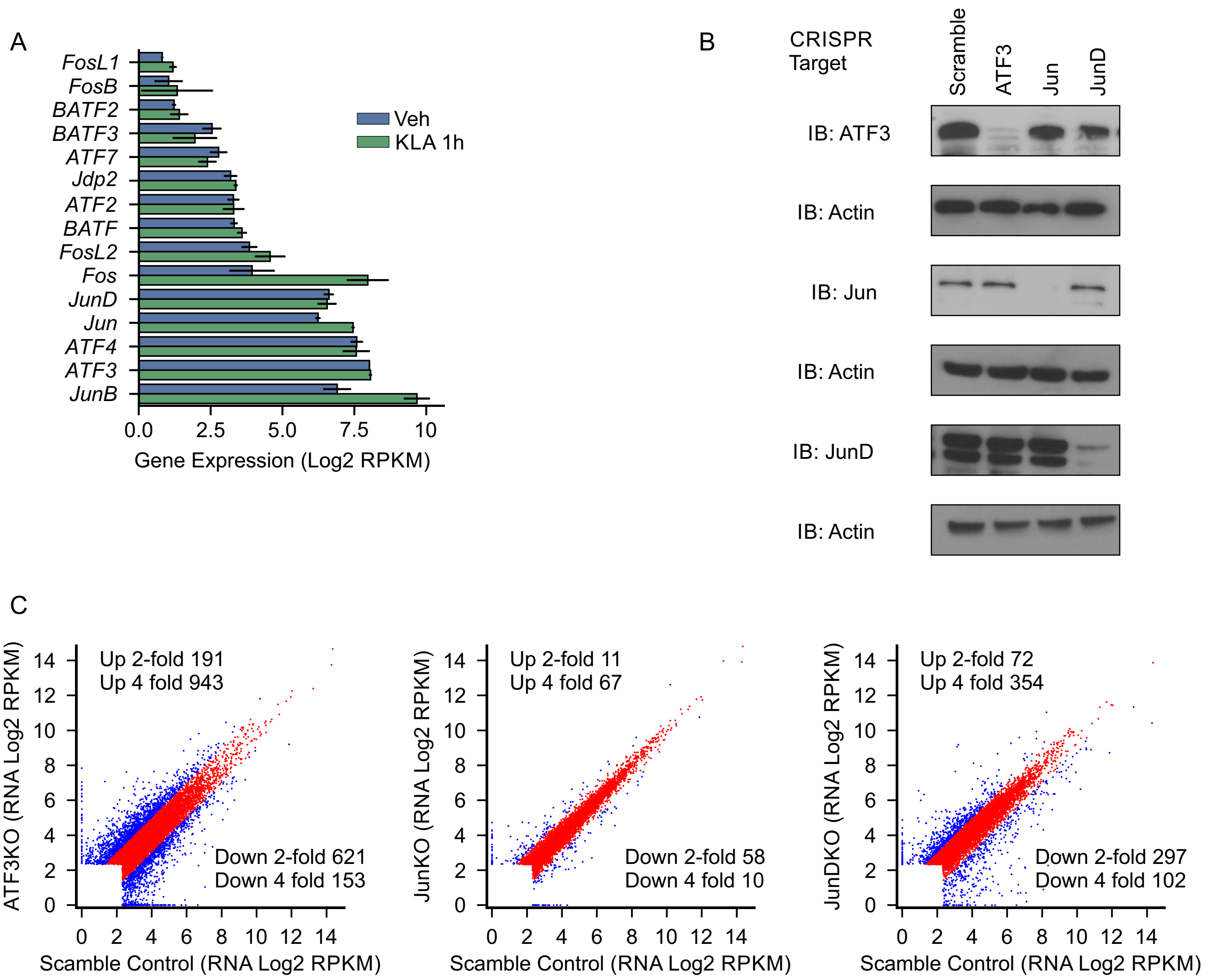

Supplementary Figure 1. A genome-wide map of AP-1 activity in macrophages. **A.** mRNA expression of all AP-1 monomers in Vehicle and 1-hour KLA treated TGEMs by RNA-seq. **B.** Western blot analysis of ATF3, Jun and JunD expression in CRISPR mediated knockout of ATF3, Jun and JunD in iBMDM cells. **C.** Scatterplots showing RNA-seq expression between scramble control and CRISPR mediated knockout of ATF3, Jun and JunD.

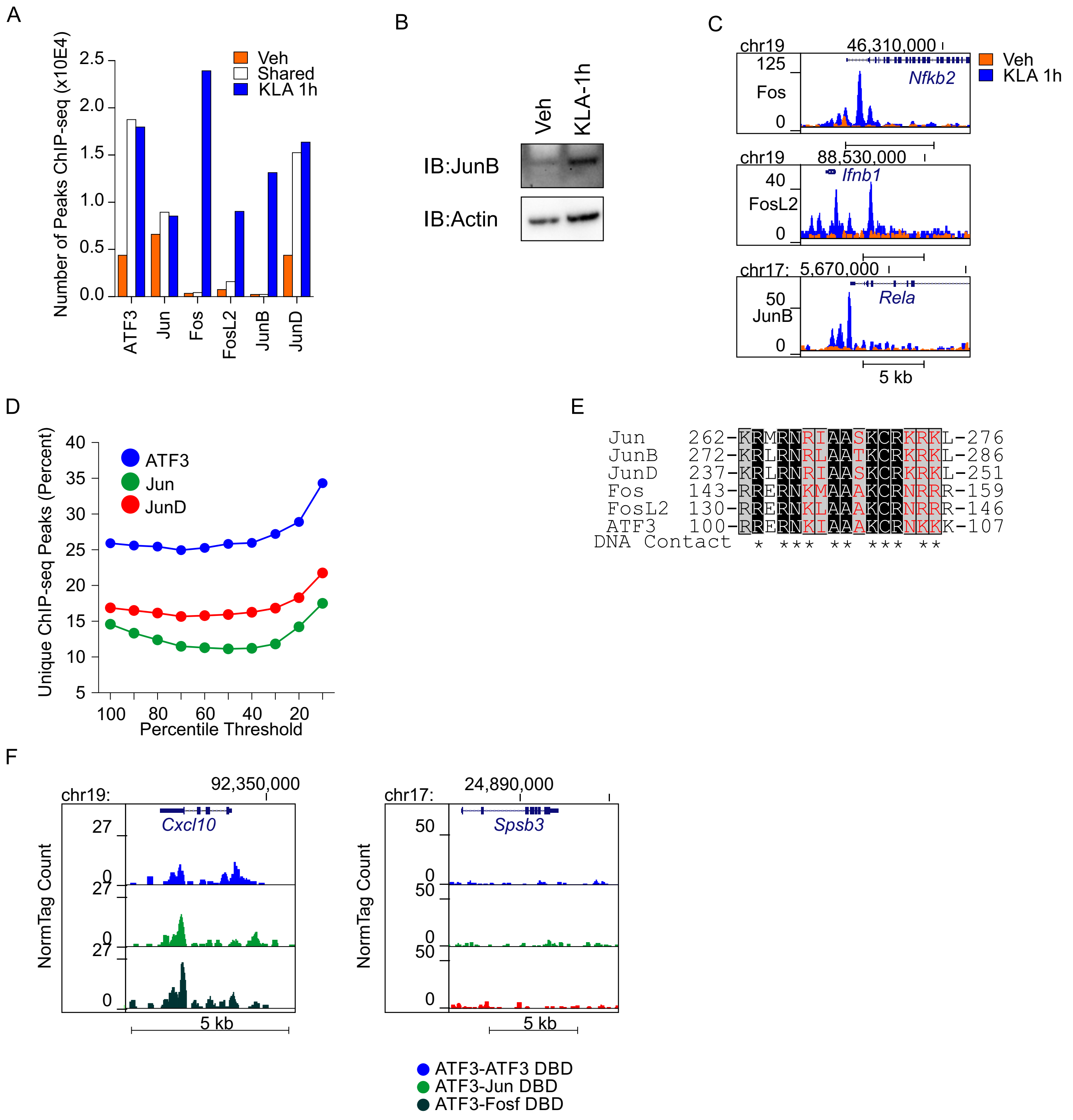

Supplementary Figure 2. Extended characterization of AP-1 binding cistrome. **A.** Quantification of the number of binding sites for each monomer that are present in vehicle, 1-hour KLA, or shared in both treatment conditions. **B.** Western blot testing the nuclear protein expression of JunB in Vehicle and KLA-1h treated TGEMs. **C.** Representative browser shots of ChIP-seq peaks for KLA specific monomers Fos (top), FosL2 (middle), and JunB (bottom). **D.** Percent of peaks that are unique to each monomer at different thresholds for the number of reads at each peak. 100 indicates 100% of the peaks and 10 indicates the top 10% of peaks when sorting by the number of reads. **E.** Protein alignment of AP-1 monomer DNA binding domains by Clustal Omega. Black background denotes identity while grey background denotes similar charge. Amino acids involved in DNA binding are indicated by stars. **F.** Representative browser shots of ChIP-seq peaks from ATF3 DNA binding domain chimeras at an ATF3 specific site (left) and Jun specific site (right).

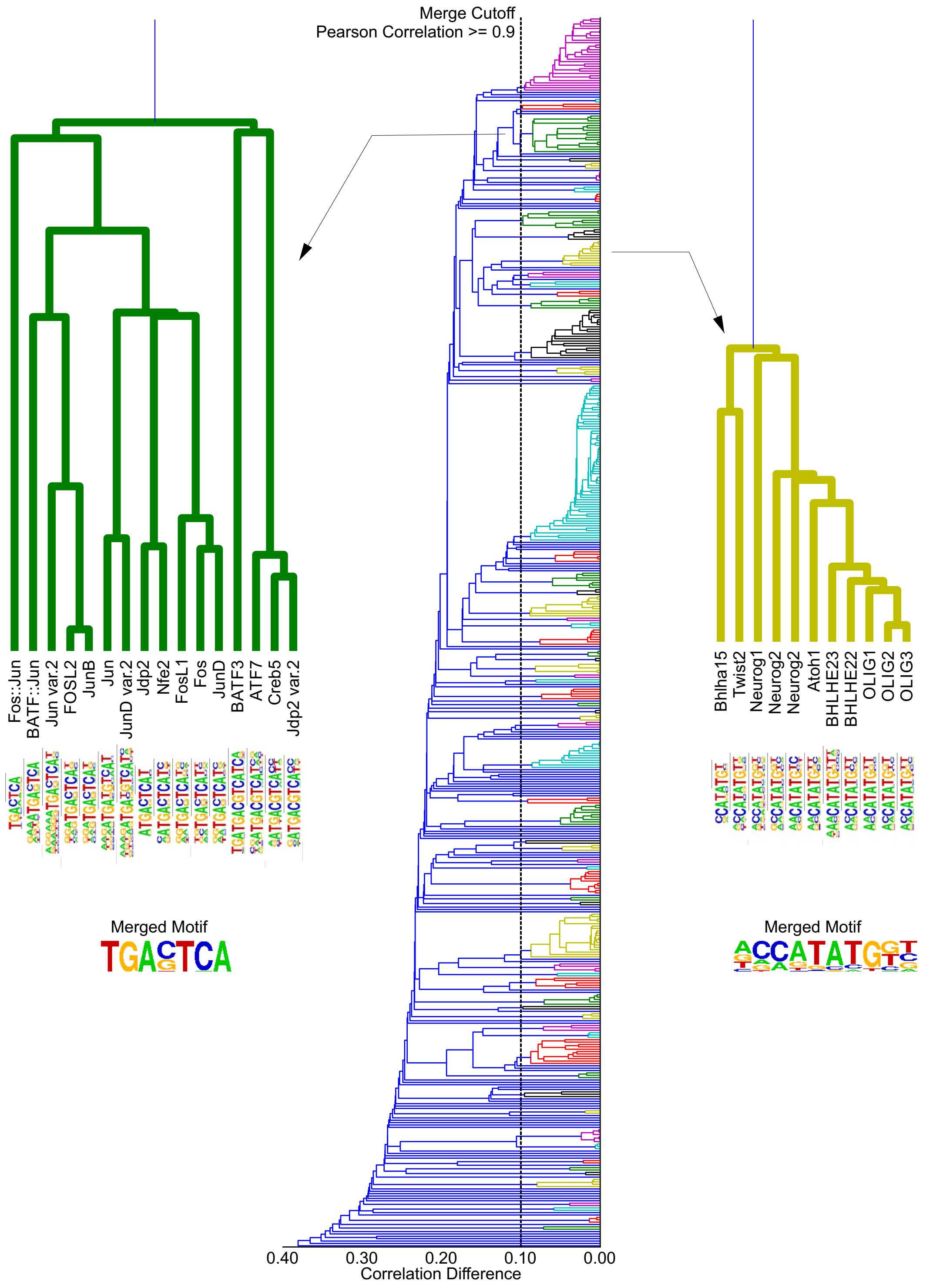

Supplementary Figure 3. Curation of the JASPAR motif library to eliminate highly similar motifs. Hierarchical clustering of all motifs in the JASPAR non-redundant library. Colored clades give highly similar motifs (Pearson Correlation ≥ 0.9, indicated by the dotted line) and blue leaf nodes give motifs that are distinct from all other motifs (Pearson Correlation < 0.9). Representative clades are indicated on the left and right (with the motif logos indicated).

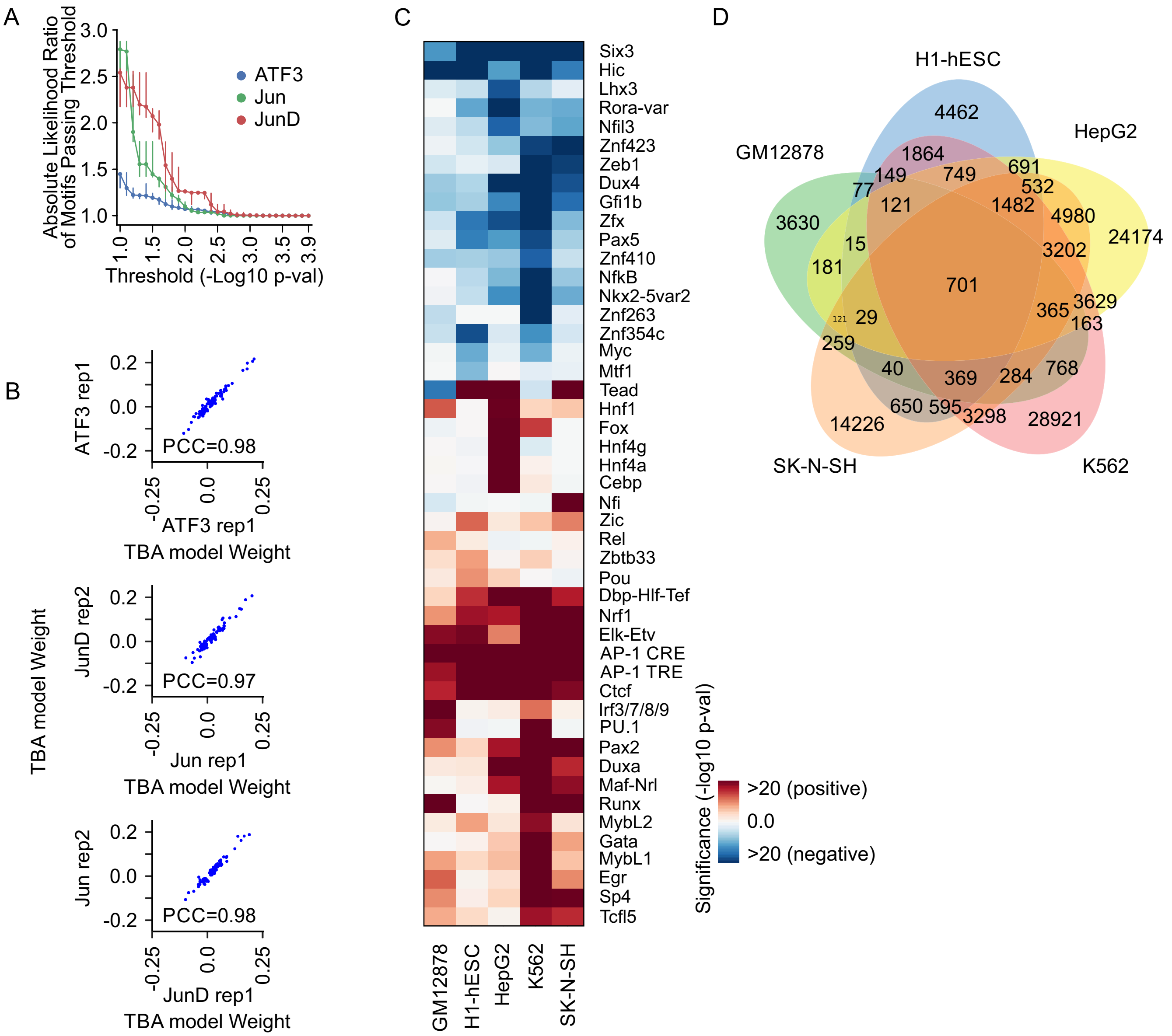

Supplementary Figure 4. Characterization of TBA on individual replicate experiments and JunD ChIP-data from different cell lines. **A.** Distribution of the absolute ratio of the likelihood value for each motif that has mean likelihood below the threshold indicated on the horizontal axis, when comparing models trained on individual replicate experiments. Likelihood values for each individual replicate are calculated using the likelihood ratio test, and then averaged across 5 cross validation sets. Error bars indicate the standard deviation. **B.** Comparison of the weights of significant motifs ($p<10e-2.5$) from TBA models calculated from individual experiments. The similarity of each pair of models, measured by the Pearson correlation coefficient, is annotated in each panel. **C.** Significance values for the 20 most significant motifs for TBA models trained for JunD binding in a several of cell lines. Red hues indicate motifs positively correlated with binding and blue hues indicate motifs negatively correlated with binding. **D.** Overlap of JunD binding sites from various cell lines. Labels indicate the number of binding sites that overlap between a combination of the cell lines.

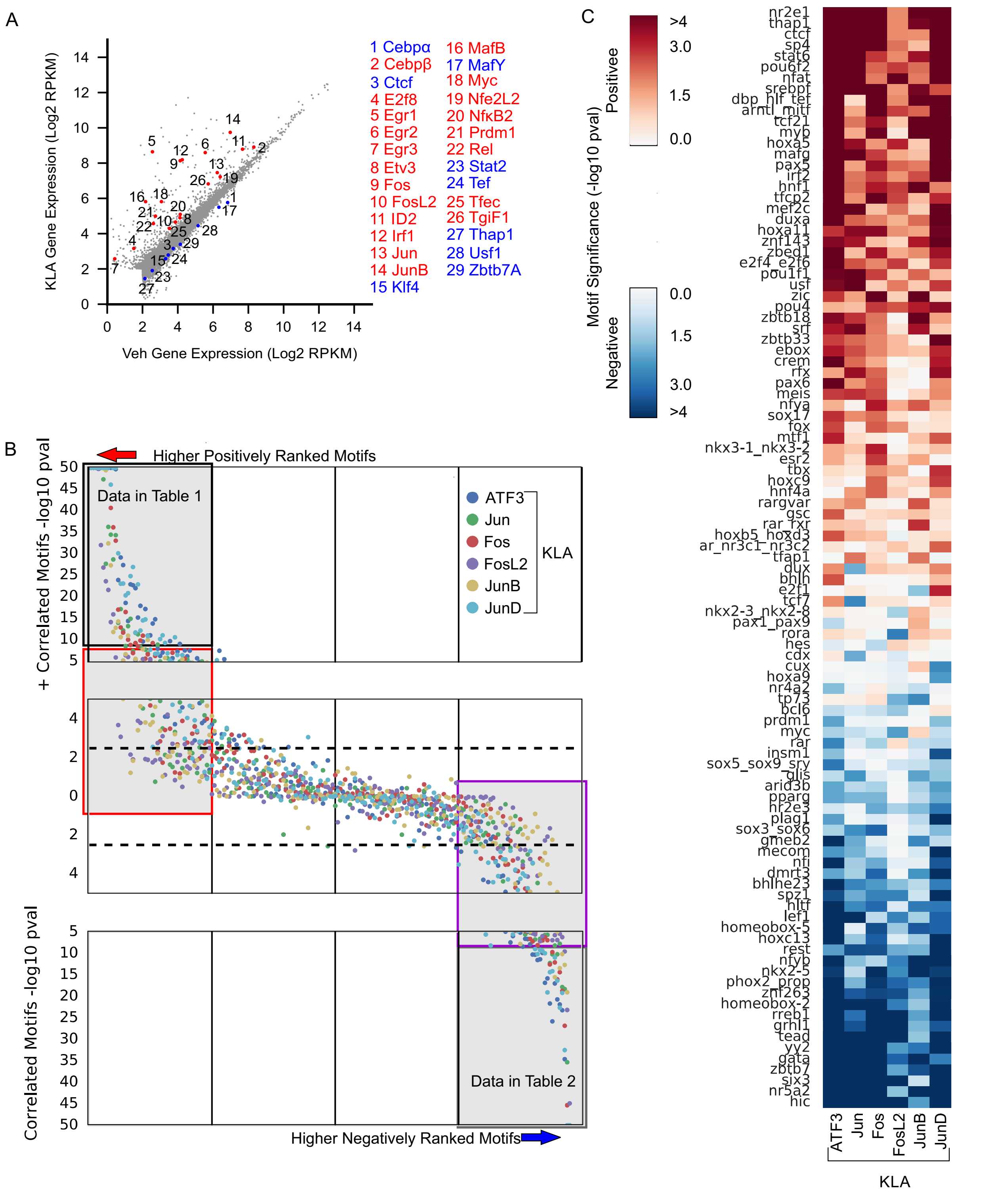

Supplementary Figure 5. TBA identifies motifs that coordinate the binding of each AP-1 monomer in KLA-1h treatment. **A.** mRNA expression of transcripts before and after KLA-1h treatment. Differentially expressed (FDR<0.05) factors with known DNA motifs are highlighted in red (up-regulated) and blue (down-regulated) , labeled and listed to the right. **B.** DNA motifs rank order based on the significance of the motif according to the likelihood ratio test. **C.** Heatmap representing the negative log10 p-value of each motif that shows a 100 fold likelihood ratio between two monomers when using the likelihood ratio test.

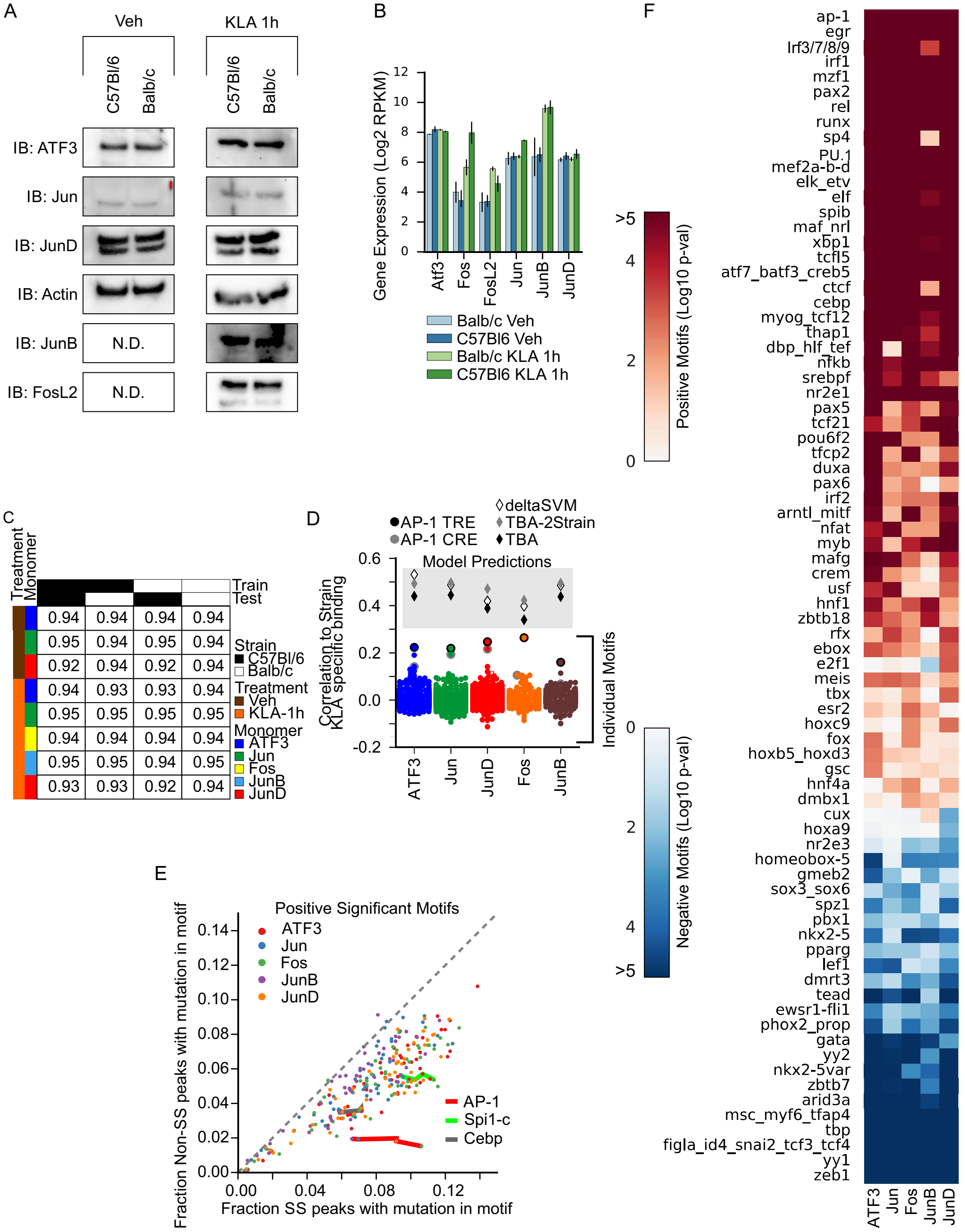
A
Veh
KLA 1h
C57Bl/6
Balb/c
C57Bl/6
Balb/c
IB: ATF3
IB: Jun
IB: JunD
IB: Actin
IB: JunB
N.D.
IB: FosL2
N.D.
B
Gene Expression (Log2 RPKM)
12
10
8
6
4
2
0
Atf3
Fos
FosL2
Jun
JunB
JunD
Balb/c Veh
C57Bl6 Veh
Balb/c KLA 1h
C57Bl6 KLA 1h
F
>5
4
2
0
Positive Motifs (Log10 p-val)
0
2
4
>5
Negative Motifs (Log10 p-val)
ap-1
egr
Irf3/7/8/9
irf1
mzf1
pax2
rel
runx
sp4
PU.1
mef2a-b-d
elk_etv
elf
spib
maf_nrl
xbp1
tcfl5
atf7_batf3_creb5
ctcf
cebp
myog_tcf12
thap1
dbp_hlf_tef
nfkb
srebpf
nr2e1
pax5
tcf21
pou6f2
tfcp2
duxa
pax6
irf2
arntl_mitf
nfat
myb
mafg
crem
usf
hnf1
zbtb18
rfx
ebox
e2f1
meis
tbx
esr2
hoxc9
fox
hoxb5_hoxd3
gsc
hnf4a
dmbx1
cux
hoxa9
nr2e3
homeobox-5
gmeb2
sox3_sox6
spz1
pbx1
nkx2-5
pparg
lef1
dmrt3
tead
ewsr1-fli1
phox2_prop
gata
yy2
nkx2-5var
zbtb7
arid3a
msc_myf6_tfap4
tbp
figla_id4_snai2_tcf3_tcf4
yy1
zeb1
ATF3
Jun
Fos
JunB
JunD
C
Treatment
Monomer
Train
Test
0.94 0.94 0.94 0.94
0.95 0.94 0.95 0.94
0.92 0.94 0.92 0.94
0.94 0.93 0.93 0.94
0.95 0.95 0.95 0.95
0.94 0.94 0.94 0.94
0.95 0.95 0.94 0.95
0.93 0.93 0.92 0.94
Strain
C57Bl/6
Balb/c
Treatment
Veh
KLA-1h
Monomer
ATF3
Jun
Fos
JunB
JunD
D
AP-1 TRE
AP-1 CRE
deltaSVM
TBA-2Strain
TBA
Model Predictions
Correlation to Strain specific binding
KLA specific binding
0.6
0.4
0.2
0.0
-0.2
Individual Motifs
ATF3
Jun
JunD
Fos
JunB
E
Positive Significant Motifs
ATF3
Jun
Fos
JunB
JunD
0.14
0.12
0.10
0.08
0.06
0.04
0.02
0.00
Fraction Non-SS peaks with mutation in motif
AP-1
Spi1-c
Cebp
0.00
0.04
0.08
0.12
Fraction SS peaks with mutation in motif

Supplementary Figure 6. Leveraging the effects of genetic variation to validate TBA predictions in activated macrophages. **A.** Western blot showing protein expression of AP-1 family members in TGEMs after Veh and one hour KLA treatment using nuclear extracts. **B.** mRNA expression of monomers before and after 1-hour KLA treatment in C57Bl/6J and BALB/cJ. **C.** Model performance when varying the strain of the data used for training and testing the TBA model. TBA was trained on AP-1 monomers in either Veh or KLA and on one strain and tested for predictive ability on either strain. **D.** Predictive performance of various models for predicting strain specific binding of each monomer as measured by the Pearson correlation of a model's predictions versus the log2 fold change in binding between the Balb/cJ and C57Bl/6J at all AP-1 binding in activated, one hour KLA treated, TGEMs. The performance of models that integrate multiple motifs – deltaSVM, TBA, TBA-2Strain, are represented as diamonds. The correlation of the change in an individual motif's score (due to a mutation) to strain specific binding is indicated using round points. **E.** Frequency of mutations in significant motifs (from TBA model, $p<10e-2.5$) at strain specific versus non-strain specific peaks for each monomer in KLA treated macrophages. **F.** Heatmap of significance values for motifs that intersected between the One Strain and Two Strain model.

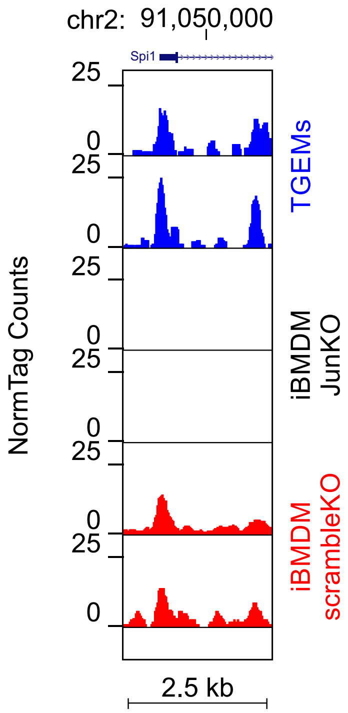

Supplementary Figure 7. CRISPR mediated knockout of Jun leads to a drastic reduction in Jun binding by ChIP-seq. ChIP-seq was performed on iBMDM where Jun knockout was performed using CRISPR, leaving 12 peaks in comparison to the 25041 detected in scramble CRISPR treated iBMDMs and 15548 in TGEMs. Representative browser shot for Jun ChIP-seq at the Spi1 locus is shown.

2 Supplementary Tables

Motif	Mean p-val
ap-1	1.00E-50
cebp	1.00E-50
spi1-c	1.00E-50
rel	1.00E-50
egr	1.15E-50
atf7_batf3_creb5	1.64E-33
pax2	2.76E-28
runx	4.26E-25
elk_etv	4.48E-23
maf_nrl	1.34E-15
irf1	2.70E-13
mzf1	1.14E-07
tcfl5	1.82E-07
spib	2.32E-07
xbp1	1.39E-06
elf	2.77E-06

Supplementary Table 1. Table of highly significant motifs, positively correlated with binding for all AP-1 monomers in KLA treated TGEMs.

Motif	Mean p-val
zeb1	2.20E-46
yy1	1.53E-20
figla_id4_snai2_tcf3_tcf4	4.78E-11
onecut	1.04E-07
tbp	3.04E-07
msc_myf6_tfap4	6.38E-07

Supplementary Table 2. Table of highly significant motifs, negatively correlated with binding for all AP-1 monomers in KLA treated TGEMs.

Reactivity	Description	Company	Cat #
ATF2	Rabbit polyclonal	Santa Cruz	sc-187
Atf3	Rabbit polyclonal	Thermo	PA5-36244
Atf4	Rabbit polyclonal	Cell Signaling	11815
Atf4	Rabbit polyclonal	Sigma	ABE387
Fos	Rabbit polyclonal	Santa Cruz	sc-7202
FosL1	Rabbit polyclonal	Santa Cruz	sc-605
FosL2	Mouse monoclonal	Santa Cruz	sc-166102
Fosb	Rabbit polyclonal	Cell Signaling	2251
Jun	Rabbit polyclonal	Santa Cruz	sc-1694
JunB	Rabbit polyclonal	Santa Cruz	sc-73
JunD	Rabbit polyclonal	Santa Cruz	sc-74
Jdp2	Rabbit polyclonal	Thermo	PA5-19692
Batf	Rabbit polyclonal	Brookwood Biomedical	PAB4003
Batf2	Rabbit polyclonal	Santa Cruz	sc-241891
Batf3	Rabbit polyclonal	Abnova	H00055509-M04
CEBPa	Rabbit polyclonal	Santa Cruz	sc-61
Pu.1	Rabbit polyclonal	Santa Cruz	sc-352
PPARg	Rabbit polyclonal	Santa Cruz	sc-7196
PPARg	Rabbit monoclonal	Cell Signaling	C26H12
PPARg	Rabbit polyclonal	Diagenode	C15410133

Supplementary Table 3. A List of Antibodies used in this study.

Target	Guide Sequence	Source
ATF3-mouse	GTCAAATACCAGTGACCCAGG	This study
ATF3-mouse	GCTTGGTGACTGACATCTCCA	This study
Jun-mouse	gcttcccagtgtcacctccg	This study
Jun-mouse	GCTCTCGGACTGGAGGAACGG	This study
JunD-mouse	gctcaggttggcgtagaccg	This study
JunD-mouse	gccgagtctcgaaagagtccg	This study
Scramble-mouse	GCACTACCAGAGCTAACTCA	This study

Supplementary Table 4. Guide RNAs used for CRISPR experiments
