# Supplementary material for: Diverse motif ensembles specify non-redundant DNA binding activities of AP-1 family members in macrophages
Source: Nat Commun. 2019 Jan 24;10:414. doi: 10.1038/s41467-018-08236-0 (PMC6345992; doi:10.1038/s41467-018-08236-0)
Supplement: Supplementary file 2 — Description of Additional Supplementary Files [file 41467_2018_8236_MOESM2_ESM.pdf]

**Description of Additional Supplementary Files**

File Name: Supplementary Data 1
Description: Source Data
