# Supplementary material for: Diverse motif ensembles specify non-redundant DNA binding activities of AP-1 family members in macrophages
Source: Nat Commun. 2019 Jan 24;10:414. doi: 10.1038/s41467-018-08236-0 (PMC6345992; doi:10.1038/s41467-018-08236-0)
Supplement: Supplementary file 4 — Reporting Summary [file 41467_2018_8236_MOESM4_ESM.pdf]

nature research

Corresponding author(s): Christopher K. Glass

Reporting Summary

Nature Research wishes to improve the reproducibility of the work that we publish. This form provides structure for consistency and transparency in reporting. For further information on Nature Research policies, see Authors & Referees and the Editorial Policy Checklist.

Statistical parameters

When statistical analyses are reported, confirm that the following items are present in the relevant location (e.g. figure legend, table legend, main text, or Methods section).

n/a	Confirmed	
☐	☒	The exact sample size (*n*) for each experimental group/condition, given as a discrete number and unit of measurement
☐	☒	An indication of whether measurements were taken from distinct samples or whether the same sample was measured repeatedly
☐	☒	The statistical test(s) used AND whether they are one- or two-sided<br>*Only common tests should be described solely by name; describe more complex techniques in the Methods section.*
☐	☒	A description of all covariates tested
☐	☒	A description of any assumptions or corrections, such as tests of normality and adjustment for multiple comparisons
☐	☒	A full description of the statistics including central tendency (e.g. means) or other basic estimates (e.g. regression coefficient) AND variation (e.g. standard deviation) or associated estimates of uncertainty (e.g. confidence intervals)
☐	☒	For null hypothesis testing, the test statistic (e.g. *F*, *t*, *r*) with confidence intervals, effect sizes, degrees of freedom and *P* value noted<br>*Give P values as exact values whenever suitable.*
☐	☒	For Bayesian analysis, information on the choice of priors and Markov chain Monte Carlo settings
☐	☒	For hierarchical and complex designs, identification of the appropriate level for tests and full reporting of outcomes
☐	☒	Estimates of effect sizes (e.g. Cohen's *d*, Pearson's *r*), indicating how they were calculated
☐	☒	Clearly defined error bars<br>*State explicitly what error bars represent (e.g. SD, SE, CI)*

Our web collection on statistics for biologists may be useful.

Software and code

Policy information about availability of computer code

Data collection	The Illumina bcl2fastq tool was used to process raw sequencing basecalls from Illumina sequencer machines (HiSeq 2500, HiSeq4000 and NextSeq 500).
Data analysis	BaMM motif and gkm-SVM were both run with default parameters. We used the latest version of the larger scale gkm-SVM, LS-GKM (compiled from source code downloaded from https://github.com/Dongwon-Lee/lsgkm on 8/25/16), and BaMM motif (v1.0 downloaded from github.com/soedinglab/BaMMmotif). Both models were trained using five-fold cross validation. Model performance was scored using roc_auc_score and precision_score functions from the metrics module of sklearn.<br><br>Other analysis were performed using Python (with the packages - numpy, pandas, sklearn, matplotlib, seaborn, scipy, Biopython). The source code for our model is available at: https://github.com/jenhantao/tba

For manuscripts utilizing custom algorithms or software that are central to the research but not yet described in published literature, software must be made available to editors/reviewers upon request. We strongly encourage code deposition in a community repository (e.g. GitHub). See the Nature Research guidelines for submitting code & software for further information.

Data

Policy information about availability of data

All manuscripts must include a data availability statement. This statement should provide the following information, where applicable:

- Accession codes, unique identifiers, or web links for publicly available datasets
- A list of figures that have associated raw data
- A description of any restrictions on data availability

Data generated for this study has been deposited to the NCBI Gene Expression Omnibus (GEO) under the accession number GSE111856

Field-specific reporting

Please select the best fit for your research. If you are not sure, read the appropriate sections before making your selection.

☒ Life sciences ☐ Behavioural & social sciences ☐ Ecological, evolutionary & environmental sciences

For a reference copy of the document with all sections, see nature.com/authors/policies/ReportingSummary-flat.pdf

Life sciences study design

All studies must disclose on these points even when the disclosure is negative.

Sample size	Sequencing experiments were done in duplicate
Data exclusions	AP-1 family members were excluded by ChIP-sequencing based on low peak counts or quality.
Replication	Reproducibility of sequencing data was performed by IDR (Irreproducibe discovery rate).
Randomization	Randomization was not relevant to this study
Blinding	Blinding was not possible for this study

Reporting for specific materials, systems and methods

Materials & experimental systems

n/a	Involved in the study	
☒	☐	Unique biological materials
☐	☒	Antibodies
☒	☐	Eukaryotic cell lines
☒	☐	Palaeontology
☐	☒	Animals and other organisms
☒	☐	Human research participants

Methods

n/a	Involved in the study	
☐	☒	ChIP-seq
☒	☐	Flow cytometry
☒	☐	MRI-based neuroimaging

Antibodies

Antibodies used

Fos Rabbit polyclonal Santa Cruz sc-7202
FosL1 Rabbit polyclonal Santa Cruz sc-605
FosL2 Mouse monoclonal Santa Cruz sc-166102
Fosb Rabbit polyclonal Cell Signaling 2251
Jun Rabbit polyclonal Santa Cruz sc-1694
Jun Mouse monoclonal Santa Cruz sc-74543
JunB Rabbit polyclonal Santa Cruz sc-73
JunD Rabbit polyclonal Santa Cruz sc-74
Jdp2 Rabbit polyclonal Thermo PA5-19692
Batf Rabbit polyclonal Brookwood Biomedical PAB4003
Batf2 Rabbit polyclonal Santa Cruz sc-241891
Batf3 Rabbit polyclonal Abnova H00055509-M04
C/ebp Rabbit polyclonal Santa Cruz sc-61
Pu.1 Rabbit polyclonal Santa Cruz sc-352

PPARg Rabbit polyclonal Santa Cruz sc-7196
PPARg Rabbit monoclonal Cell Signaling C26H12
PPARg Rabbit polyclonal Diagenode C15410133

Validation

All antibodies were tested by the manufacturers using Western Blot, ChIP, or both.

Methods for each specific antibody are as follows:
Reactivity,Description,Company,Catalog #, Validation Method
ATF2,Rabbit polyclonal,Santa Cruz,sc-187,ChIP
Atf3,Rabbit polyclonal,Thermo,PA5-41308,Western
Atf3,Rabbit polyclonal,Thermo,PA5-36244,ChIP
Atf4,Rabbit polyclonal,Cell Signaling,11815,ChIP
Atf4,Rabbit polyclonal,Sigma,ABE387,ChIP
Fos,Rabbit polyclonal,Santa Cruz,sc-7202,Western/ChIP
FosL1,Rabbit polyclonal,Santa Cruz,sc-605,ChIP
FosL2,Mouse monoclonal,Santa Cruz,sc-166102,Western/ChIP
Fosb,Rabbit polyclonal,Cell Signaling,2251,ChIP
Jun,Rabbit polyclonal,Santa Cruz,sc-1694,ChIP
Jun,Mouse monoclonal,Santa Cruz,sc-74543,Western
JunB,Rabbit polyclonal,Santa Cruz,sc-73,Western/ChIP
JunD,Rabbit polyclonal,Santa Cruz,sc-74,Western/ChIP
Jdp2,Rabbit polyclonal,Thermo,PA5-19692,ChIP
Batf,Rabbit polyclonal,Brookwood Biomedical,PAB4003,ChIP
Batf2,Rabbit polyclonal,Santa Cruz,sc-241891,ChIP
Batf3,Rabbit polyclonal,Abnova,H00055509-M04,ChIP
C/ebp,Rabbit polyclonal,Santa Cruz,sc-61,ChIP
Pu.1,Rabbit polyclonal,Santa Cruz,sc-352,ChIP
PPARg,Rabbit polyclonal,Santa Cruz,sc-7196,ChIP/IP
PPARg,Rabbit monoclonal,Cell Signaling,C26H12,ChIP/IP
PPARg,Rabbit polyclonal,Diagenode,C15410133,ChIP/IP

Animals and other organisms

Policy information about studies involving animals; ARRIVE guidelines recommended for reporting animal research

Laboratory animals

C57Bl/6EiJ/ Male/ 8-10 weeks; Balbc/J/Male/8-10 weeks/ ; PPARgamma-MXCre-lysMCre(

Wild animals

No wild animals were used for this study

Field-collected samples

No field collected samples were used for this study

ChIP-seq

Data deposition

☒ Confirm that both raw and final processed data have been deposited in a public database such as GEO.

☒ Confirm that you have deposited or provided access to graph files (e.g. BED files) for the called peaks.

Data access links
May remain private before publication.

https://www.ncbi.nlm.nih.gov/geo/query/acc.cgi?acc=GSE111856

Files in database submission

ChIP-seq Files:
Balbc_Thiomac_ChIP_ATF3_KLA-1h_GJF_16-07-25.fastq.gz
Balbc_Thiomac_ChIP_ATF3_KLA-1h_GJF_16-08-16-rep1.fastq.gz
Balbc_Thiomac_ChIP_ATF3_Veh_GJF_16-08-16.fastq.gz
Balbc_Thiomac_ChIP_ATF3_Veh_GJF_16-08-16-rep1.fastq.gz
Balbc_Thiomac_ChIP_cJun_KLA-1h_GFEW_10-19-15.fastq.gz
Balbc_Thiomac_ChIP_cJun_KLA-1h_GJF_16-06-12.fastq.gz
Balbc_Thiomac_ChIP_cJun_Veh_GFEW_10-19-15.fastq.gz
Balbc_Thiomac_ChIP_cJun_Veh_GJF_16-06-12.fastq.gz
Balbc_Thiomac_ChIP_Fos_KLA-1h_GFEW_10-19-15.fastq.gz
Balbc_Thiomac_ChIP_Fos_KLA-1h_GJF_16-07-22.fastq.gz
Balbc_Thiomac_ChIP_Fos_Veh_GFEW_10-19-15.fastq.gz
Balbc_Thiomac_ChIP_Fos_Veh_GJF_16-07-22.fastq.gz
Balbc_Thiomac_ChIP_Input_KLA-1h_GJF_15-03-20.fastq.gz
Balbc_Thiomac_ChIP_Input_Veh_GJF_15-03-20.fastq.gz
Balbc_Thiomac_ChIP_JunB_KLA-1h_GFEW_10-19-15.fastq.gz
Balbc_Thiomac_ChIP_JunB_KLA-1h_GJF_16-10-13.fastq.gz
Balbc_Thiomac_ChIP_JunB_Veh_GFEW_10-19-15.fastq.gz
Balbc_Thiomac_ChIP_JunB_Veh_GJF_16-07-22.fastq.gz
Balbc_Thiomac_ChIP_JunD_KLA-1h_GFEW_10-19-15.fastq.gz

Balbc_Thiomac_ChIP_JunD_KLA-1h_GJF_16-10-13.fastq.gz
Balbc_Thiomac_ChIP_JunD_Veh_GFEW_10-19-15.fastq.gz
Balbc_Thiomac_ChIP_JunD_Veh_GJF_16-06-12.fastq.gz
C57Bl6-PPARgKO_Thiomac_ChIP_ATF3_Veh_GJF_16-07-23-1.fastq.gz
C57Bl6-PPARgKO_Thiomac_ChIP_ATF3_Veh_GJF_16-07-23-2.fastq.gz
C57Bl6-PPARgKO_Thiomac_ChIP_cJun_Veh_GJF_16-07-23-1.fastq.gz
C57Bl6-PPARgKO_Thiomac_ChIP_cJun_Veh_GJF_16-07-23-2.fastq.gz
C57Bl6-PPARgKO_Thiomac_ChIP_JunD_Veh_GJF_16-07-23-1.fastq.gz
C57Bl6-PPARgKO_Thiomac_ChIP_JunD_Veh_GJF_16-07-23-2.fastq.gz
C57Bl6_Thiomac_ChIP_ATF3_KLA-1h_GJF_16-04-21.fastq.gz
C57Bl6_Thiomac_ChIP_ATF3_KLA-1h_GJF_16-08-16.fastq.gz
C57Bl6_Thiomac_ChIP_ATF3_Veh_GJF_16-04-21.fastq.gz
C57Bl6_Thiomac_ChIP_ATF3_Veh_GJF_16-07-23.fastq.gz
C57Bl6_Thiomac_ChIP_CEBPa_KLA-1h_GFEW_15-11-18.fastq.gz
C57Bl6_Thiomac_ChIP_CEBPa_Veh_GJF_16-06-14.fastq.gz
C57Bl6_Thiomac_ChIP_cJun_KLA-1h_GFEW_15-11-18.fastq.gz
C57Bl6_Thiomac_ChIP_cJun_KLA-1h_GJF_16-04-21.fastq.gz
C57Bl6_Thiomac_ChIP_cJun_Veh_GJF_16-04-21.fastq.gz
C57Bl6_Thiomac_ChIP_cJun_Veh_GJF_16-06-14.fastq.gz
C57Bl6_Thiomac_ChIP_Fos_KLA-1h_GJF_14-03-17.fastq.gz
C57Bl6_Thiomac_ChIP_Fos_KLA-1h_GJF_15-02-06.fastq.gz
C57Bl6_Thiomac_ChIP_Fos_Veh_GJF_14-03-17.fastq.gz
C57Bl6_Thiomac_ChIP_Fos_Veh_GJF_15-02-06.fastq.gz
C57Bl6_Thiomac_ChIP_Fra2_KLA-1h_GJF_16-04-21.fastq.gz
C57Bl6_Thiomac_ChIP_Fra2_KLA-1h_GJF_16-08-16.fastq.gz
C57Bl6_Thiomac_ChIP_Fra2_Veh_GJF_14-03-17.fastq.gz
C57Bl6_Thiomac_ChIP_Fra2_Veh_GJF_16-04-21.fastq.gz
C57Bl6_Thiomac_ChIP_Input_KLA-1h_GJF_15-03-20.fastq.gz
C57Bl6_Thiomac_ChIP_Input_Veh_GJF_15-03-20.fastq.gz
C57Bl6_Thiomac_ChIP_JunB_KLA-1h_GJF_14-03-17.fastq.gz
C57Bl6_Thiomac_ChIP_JunB_KLA-1h_GJF_15-02-06.fastq.gz
C57Bl6_Thiomac_ChIP_JunB_Veh_GJF_15-02-06.fastq.gz
C57Bl6_Thiomac_ChIP_JunB_Veh_GJF_16-04-21.fastq.gz
C57Bl6_Thiomac_ChIP_JunD_KLA-1h_GFEW_15-11-18.fastq.gz
C57Bl6_Thiomac_ChIP_JunD_KLA-1h_GJF_16-04-21.fastq.gz
C57Bl6_Thiomac_ChIP_JunD_Veh_GJF_16-04-21.fastq.gz
C57Bl6_Thiomac_ChIP_JunD_Veh_GJF_16-07-23.fastq.gz
C57Bl6_Thiomac_ChIP_PPARg_Veh_NJS_13-04-03-1.fastq.gz
C57Bl6_Thiomac_ChIP_PPARg_Veh_NJS_13-04-03-2.fastq.gz
C57Bl6_Thiomac_ChIP_PU1_KLA-1h_TKS_12-01-14.fastq.gz
C57Bl6_Thiomac_ChIP_PU1_Veh_GJF_16-06-14.fastq.gz
C57Bl6_iBMDM_ChIP_Jun_negscr_rep1_GJF_18-02-18-rep1.fastq.gz
C57Bl6_iBMDM_ChIP_Jun_negscr_rep1_GJF_18-02-18-rep2.fastq.gz
C57Bl6-Atf3KO-Atf3DBD_iBMDM_ChIP_Veh_GJF_18-02-20-rep1.fastq
C57Bl6-Atf3KO-Atf3DBD_iBMDM_ChIP_Veh_GJF_18-02-20-rep2.fastq
C57Bl6-FosKO-Atf3DBD_iBMDM_ChIP_Veh_GJF_18-02-20-rep1.fastq
C57Bl6-FosKO-Atf3DBD_iBMDM_ChIP_Veh_GJF_18-02-20-rep2.fastq
C57Bl6-JunKO-Atf3DBD_iBMDM_ChIP_Veh_GJF_18-02-20-rep1.fastq
C57Bl6-JunKO-Atf3DBD_iBMDM_ChIP_Veh_GJF_18-02-20-rep2.fastq

balbc_atf3_kla_peaks.tsv
balbc_atf3_veh_peaks.tsv
balbc_cjun_kla_peaks.tsv
balbc_cjun_veh_peaks.tsv
balbc_fos_kla_peaks.tsv
balbc_fos_veh_peaks.tsv
balbc_junb_kla_peaks.tsv
balbc_junb_veh_peaks.tsv
balbc_jund_kla_peaks.tsv
balbc_jund_veh_peaks.tsv
c57bl6_atf3_kla_peaks.tsv
c57bl6_atf3_veh_peaks.tsv
c57bl6_cebpa_kla_peaks.tsv
c57bl6_cebpa_veh_peaks.tsv
c57bl6_cjun_kla_peaks.tsv
c57bl6_cjun_veh_peaks.tsv
c57bl6_cjun_negscr_peaks.tsv
c57bl6_fos_kla_peaks.tsv
c57bl6_fos_veh_peaks.tsv
c57bl6_fra2_kla_peaks.tsv
c57bl6_fra2_veh_peaks.tsv
c57bl6_junb_kla_peaks.tsv
c57bl6_junb_veh_peaks.tsv
c57bl6_jund_kla_peaks.tsv
c57bl6_jund_veh_peaks.tsv

c57bl6-ppargko_atf3_veh_peaks.tsv
c57bl6-ppargko_cjun_veh_peaks.tsv
c57bl6-ppargko_jund_veh_peaks.tsv
c57bl6_pparg_veh_peaks.tsv
c57bl6_pu1_kla_peaks.tsv
c57bl6_pu1_veh_peaks.tsv
C57Bl6_iBMDM_ChIP_Jun_negscr_peaks.tsv
C57Bl6-Atf3KO-Atf3DBD_iBMDM_ChIP_Veh_peaks.tsv
C57Bl6-FosKO-Atf3DBD_iBMDM_ChIP_Veh_peaks.tsv
C57Bl6-JunKO-Atf3DBD_iBMDM_ChIP_Veh_peaks.tsv

Genome browser session
(e.g. UCSC)

https://genome.ucsc.edu/cgi-bin/hgTracks?db=mm10&lastVirtModeType=default&lastVirtModeExtraState=&virtModeType=default&virtMode=0&nonVirtPosition=&position=chr2%3A91111792%2D91130873&hgsid=691425423_PcaidXtMcJWKMDrZtVhQbx5ANm3f

Methodology

Replicates

ChIP-seq experiments were performed using two biological replicates.

Sequencing depth

All experiments were sequenced using single-end sequencing with 50 basepair reads. Sequencing depth information is as follows:
sample,totalReads,uniquelyMappedReads
C57Bl6_Thiomac_ChIP_ATF3_KLA-1h_GJF_16-04-21,20832779,15944758
C57Bl6_Thiomac_ChIP_ATF3_KLA-1h_GJF_16-08-16,17798440,14851431
C57Bl6_Thiomac_ChIP_ATF3_Veh_GJF_16-04-21,13129371,9934499
C57Bl6_Thiomac_ChIP_ATF3_Veh_GJF_16-07-23,21304609,16430542
C57Bl6_Thiomac_ChIP_CEBPa_KLA-1h_GFEW_15-11-18,13195714,9503237
C57Bl6_Thiomac_ChIP_CEBPa_KLA-1h_SH_11-05-12,9980924,7225599
C57Bl6_Thiomac_ChIP_CEBPa_Veh_GJF_16-06-14,23465571,16824200
C57Bl6_Thiomac_ChIP_CEBPa_Veh_SH_11-05-12,10388710,7372737
C57Bl6_Thiomac_ChIP_cJun_KLA-1h_GFEW_15-11-18,15636115,11756873
C57Bl6_Thiomac_ChIP_cJun_KLA-1h_GJF_16-04-21,13983075,10318023
C57Bl6_Thiomac_ChIP_cJun_Veh_GJF_16-04-21,27666255,18428224
C57Bl6_Thiomac_ChIP_cJun_Veh_GJF_16-06-14,21121665,14330648
C57Bl6_Thiomac_ChIP_Fos_KLA-1h_GJF_14-03-17,15703117,11193102
C57Bl6_Thiomac_ChIP_Fos_KLA-1h_GJF_15-02-06,22568433,17878543
C57Bl6_Thiomac_ChIP_Fos_Veh_GJF_14-03-17,15022156,2161299
C57Bl6_Thiomac_ChIP_Fos_Veh_GJF_15-02-06,23861304,17304730
C57Bl6_Thiomac_ChIP_Fra2_KLA-1h_GJF_16-04-21,18582476,14149096
C57Bl6_Thiomac_ChIP_Fra2_KLA-1h_GJF_16-08-16,17430209,13151872
C57Bl6_Thiomac_ChIP_Fra2_Veh_GJF_14-03-17,13001175,7281422
C57Bl6_Thiomac_ChIP_Fra2_Veh_GJF_16-04-21,11047282,8338487
C57Bl6_Thiomac_ChIP_JunB_KLA-1h_GJF_14-03-17,12750365,4348064
C57Bl6_Thiomac_ChIP_JunB_KLA-1h_GJF_15-02-06,17860142,13755939
C57Bl6_Thiomac_ChIP_JunB_Veh_GJF_15-02-06,20196779,15122749
C57Bl6_Thiomac_ChIP_JunB_Veh_GJF_16-04-21,27885982,19626709
C57Bl6_Thiomac_ChIP_JunD_KLA-1h_GFEW_15-11-18,7574382,5763541
C57Bl6_Thiomac_ChIP_JunD_KLA-1h_GJF_16-04-21,19651259,15292546
C57Bl6_Thiomac_ChIP_JunD_Veh_GJF_16-04-21,25347922,19560902
C57Bl6_Thiomac_ChIP_JunD_Veh_GJF_16-07-23,25115568,19084430
C57Bl6_Thiomac_ChIP_p65_KLA-1h_CR_12-11-08-2,16717962,14404386
C57Bl6_Thiomac_ChIP_p65_KLA-1h_KA_13-06-11-3M,17182495,15467773
C57Bl6_Thiomac_ChIP_p65_Veh_CR_12-11-08-3,16122228,11209826
C57Bl6_Thiomac_ChIP_p65_Veh_SH_11-07-21,15743452,7815895
C57Bl6_Thiomac_ChIP_PU1_KLA-1h_SH_11-05-12,9778913,7935541
C57Bl6_Thiomac_ChIP_PU1_KLA-1h_TKS_12-01-14,5542090,4050035
C57Bl6_Thiomac_ChIP_PU1_Veh_GJF_16-06-14,84806668,66392795
C57Bl6_Thiomac_ChIP_PU1_Veh_SH_11-05-12,11587708,9388704
Balbc_Thiomac_ChIP_ATF3_KLA-1h_GJF_16-07-25,13776222,11269562
Balbc_Thiomac_ChIP_ATF3_KLA-1h_GJF_16-08-16-rep1,14365243,11103411
Balbc_Thiomac_ChIP_ATF3_Veh_GJF_16-08-16,21450044,16933740
Balbc_Thiomac_ChIP_ATF3_Veh_GJF_16-08-16-rep1,12911093,9752228
Balbc_Thiomac_ChIP_cJun_KLA-1h_GFEW_10-19-15,8663960,6070297
Balbc_Thiomac_ChIP_cJun_KLA-1h_GJF_16-06-12,15496322,11434865
Balbc_Thiomac_ChIP_cJun_Veh_GFEW_10-19-15,14430555,8818751
Balbc_Thiomac_ChIP_cJun_Veh_GJF_16-06-12,16263270,11821438
Balbc_Thiomac_ChIP_Fos_KLA-1h_GFEW_10-19-15,14690341,12535555
Balbc_Thiomac_ChIP_Fos_KLA-1h_GJF_16-07-22,27427559,21813100
Balbc_Thiomac_ChIP_Fos_Veh_GFEW_10-19-15,14584201,9306290
Balbc_Thiomac_ChIP_Fos_Veh_GJF_16-07-22,25285796,19751691
Balbc_Thiomac_ChIP_Fra2_KLA-1h_GJF_17-04-17-rep1,25418723,17807355
Balbc_Thiomac_ChIP_Fra2_KLA-1h_GJF_17-04-17-rep2,33366423,23317968
Balbc_Thiomac_ChIP_Fra2_Veh_GJF_17-04-17-rep1,29349166,20643605
Balbc_Thiomac_ChIP_Fra2_Veh_GJF_17-04-17-rep2,18338159,12625068
balbc_Thiomac_ChIP_Input_KLA-1h_GJF_15-03-20,18091532,14010523

balbc_Thiomac_ChIP_Input_Veh_GJF_15-03-20,19163529,14755838
Balbc_Thiomac_ChIP_JunB_KLA-1h_GFEW_10-19-15,10699475,7957973
Balbc_Thiomac_ChIP_JunB_KLA-1h_GJF_16-10-13,33235992,21246092
Balbc_Thiomac_ChIP_JunB_Veh_GFEW_10-19-15,16893268,12257203
Balbc_Thiomac_ChIP_JunB_Veh_GJF_16-07-22,36659682,21313858
Balbc_Thiomac_ChIP_JunD_KLA-1h_GFEW_10-19-15,23177438,18589699
Balbc_Thiomac_ChIP_JunD_KLA-1h_GJF_16-10-13,16793235,12392873
Balbc_Thiomac_ChIP_JunD_Veh_GFEW_10-19-15,17053352,13166237
Balbc_Thiomac_ChIP_JunD_Veh_GJF_16-06-12,18132403,13994506
Balbc_Thiomac_ChIP_p65_KLA-1h_GJF_16-07-25-rep1,23411560,18636794
Balbc_Thiomac_ChIP_p65_KLA-1h_GJF_16-08-16,18893355,14639153
Balbc_Thiomac_ChIP_p65_Veh_GJF_16-07-22-rep1,20011412,14203439
Balbc_Thiomac_ChIP_p65_Veh_GJF_16-07-22-rep2,22364065,17296430
Balbc_Thiomac_ChIP_PU1_KLA-1h_GFEW_10-19-15,51135494,42271859
Balbc_Thiomac_ChIP_PU1_KLA-1h_GJF_16-06-12,11670965,8831119
Balbc_Thiomac_ChIP_PU1_Veh_GFEW_10-19-15,22315429,17907846
Balbc_Thiomac_ChIP_PU1_Veh_GJF_16-06-12,15152742,12503148
C57Bl6-negAtf3KOAtf3DBD_iBMDM_ChIP_ATF3_Veh_GJF_18-02-20-rep1,23194439,14854197
C57Bl6-negAtf3KOAtf3DBD_iBMDM_ChIP_ATF3_Veh_GJF_18-02-20-rep2,12585875,8522448
C57Bl6-negAtf3KOFosDBD_iBMDM_ChIP_ATF3_Veh_GJF_18-02-20-rep1,12998513,8788737
C57Bl6-negAtf3KOFosDBD_iBMDM_ChIP_ATF3_Veh_GJF_18-02-20-rep2,13953195,9190279
C57Bl6-negAtf3KOJunDBD_iBMDM_ChIP_ATF3_Veh_GJF_18-02-20-rep1,14775525,10263483
C57Bl6-negAtf3KOJunDBD_iBMDM_ChIP_ATF3_Veh_GJF_18-02-20-rep2,19700906,13170127
C57Bl6-PPARgKO_Thiomac_ChIP_ATF3_Veh_GJF_16-07-23-1,17657968,13234402
C57Bl6-PPARgKO_Thiomac_ChIP_ATF3_Veh_GJF_16-07-23-2,20526757,15981630
C57Bl6-PPARgKO_Thiomac_ChIP_cJun_Veh_GJF_16-07-23-2,19364962,13422113
C57Bl6-PPARgKO_Thiomac_ChIP_cJun_Veh_GJF_16-08-16,7057133,4813601
C57Bl6-PPARgKO_Thiomac_ChIP_Fos_Veh_GJF_16-07-23-1,24672506,17633503
C57Bl6-PPARgKO_Thiomac_ChIP_Fos_Veh_GJF_16-07-23-2,17824003,12828075
C57Bl6-PPARgKO_Thiomac_ChIP_JunD_Veh_GJF_16-07-23-1,19775947,14657301
C57Bl6-PPARgKO_Thiomac_ChIP_JunD_Veh_GJF_16-07-23-2,23312576,18219803
C57Bl6_Thiomac_ChIP_PPARg_Veh_NJS_13-04-03-1,21860144,15126901
C57Bl6_Thiomac_ChIP_PPARg_Veh_NJS_13-04-03-2,18484526,13443728
C57Bl6-JunKO-Atf3DBD_iBMDM_ChIP_Veh_GJF_18-02-20-rep1,12013556,8365475
C57Bl6-JunKO-Atf3DBD_iBMDM_ChIP_Veh_GJF_18-02-20-rep2,20769247,15335656

Antibodies

Fos Rabbit polyclonal Santa Cruz sc-7202
FosL1 Rabbit polyclonal Santa Cruz sc-605
FosL2 Mouse monoclonal Santa Cruz sc-166102
Fosb Rabbit polyclonal Cell Signaling 2251
Jun Rabbit polyclonal Santa Cruz sc-1694
Jun Mouse monoclonal Santa Cruz sc-74543
JunB Rabbit polyclonal Santa Cruz sc-73
JunD Rabbit polyclonal Santa Cruz sc-74
Jdp2 Rabbit polyclonal Thermo PA5-19692
Batf Rabbit polyclonal Brookwood Biomedical PAB4003
Batf2 Rabbit polyclonal Santa Cruz sc-241891
Batf3 Rabbit polyclonal Abnova H00055509-M04
C/ebp Rabbit polyclonal Santa Cruz sc-61
Pu.1 Rabbit polyclonal Santa Cruz sc-352
PPARg Rabbit polyclonal Santa Cruz sc-7196
PPARg Rabbit monoclonal Cell Signaling C26H12
PPARg Rabbit polyclonal Diagenode C15410133

Peak calling parameters

eads from each ChIP-seq sample were aligned to the mm10 build of the mouse genome using bowtie2 (with default parameters).
Sam files were converted to tag directories with HOMER
Peaks were called with HOMER with the findPeaks command using the corresponding input file and the parameters -L 0 -C 0 -fdr 0.9
HOMER peak scores from peak files corresponding to replicate experiments were used as input for IDR; peaks with IDR < 0.05 were retained
Genome_build: mm10
Supplementary_files_format_and_content: tab delimited file giving the genomic coordinates of putative binding site for each ChIP target. The "count" column indicates the HOMER peak score

Data quality

Peaks were filtered out by calculating the Irreproducible Discovery Rate (using the HOMER peak score as input for IDR). We filtered away all peaks that had IDR >=0.05.

Software

HOMER was used to perform peak calling (http://homer.ucsd.edu/homer/)

The IDR program was used to calculate IDR scores (https://github.com/nboley/idr)
